# Structural and Ultrastructural Analysis of the Cervical Discs of Young and Elderly Humans

**DOI:** 10.1371/journal.pone.0139283

**Published:** 2015-10-01

**Authors:** Ricardo Braganca de Vasconcellos Fontes, Josemberg Silva Baptista, Said Rahnamaye Rabbani, Vincent C. Traynelis, Edson Aparecido Liberti

**Affiliations:** 1 Department of Anatomy, Instituto de Ciencias Biomedicas, Universidade de Sao Paulo, Sao Paulo, SP, Brazil; 2 Department of Neurosurgery, Rush University Medical Center, Chicago, IL, United States of America; 3 Department of Morphology, Universidade Federal do Espirito Santo, Vitoria, ES, Brazil; 4 Department of General Physics, Instituto de Fisica, Universidade de Sao Paulo, Sao Paulo, SP, Brazil; Georgia Regents University, UNITED STATES

## Abstract

Several studies describing the ultrastructure and extracellular matrix (ECM) of intervertebral discs (IVDs) involve animal models and specimens obtained from symptomatic individuals during surgery for degenerative disease or scoliosis, which may not necessarily correlate to changes secondary to normal aging in humans. These changes may also be segment-specific based on different load patterns throughout life. Our objective was to describe the ECM and collagen profile of cervical IVDs in young (G1 - <35 years) and elderly (G2 - >65 years) presumably-asymptomatic individuals. Thirty cervical discs per group were obtained during autopsies of presumably-asymptomatic individuals. IVDs were analyzed with MRI, a morphological grading scale, light microscopy, scanning electron microscopy (SEM) and immunohistochemistry (IHC) for collagen types I, II, III, IV, V, VI, IX and X. Macroscopic degenerative features such as loss of annulus-nucleus distinction and fissures were found in both groups and significantly more severe in G2 as expected. MRI could not detect all morphological changes when compared even with simple morphological inspection. The loose fibrocartilaginous G1 matrix was replaced by a denser ECM in G2 with predominantly cartilaginous characteristics, chondrocyte clusters and absent elastic fibers. SEM demonstrated persistence of an identifiable nucleus and Sharpey-type insertion of cervical annulus fibers even in highly-degenerated G2 specimens. All collagen types were detected in every disc sector except for collagen X, with the largest area stained by collagens II and IV. Collagen detection was significantly decreased in G2: although significant intradiscal differences were rare, changes may occur faster or earlier in the posterior annulus. These results demonstrate an extensive modification of the ECM with maintenance of basic ultrastructural features despite severe macroscopic degeneration. Collagen analysis supports there is not a “pathologic” collagen type and changes are generally similar throughout the disc. Understanding the collagen and ultrastructural substrate of degenerative changes in the human disc is an essential step in planning restorative therapies.

## Introduction

The basic structure of the human intervertebral disc (IVD) has been known since at least 1858, while the first studies concerning the morphological changes secondary to aging (i.e., disc degeneration) date from the 1920s [1,2]. Macroscopic modifications of human IVDs related to aging, such as disappearance of vascular channels, annular fissures, osteophyte formation and ingrowth of blood vessels into the annulus fibrosus (AF) had been described by 1950, as well as an expected sequence of degenerative events, all thought to be precipitated by the largely avascular nature of the human IVD. Lumbar discs have been the main object of these studies, with only a small fraction involving cervical discs.

Countless subsequent studies have analyzed different microscopic and molecular aspects of disc degeneration but a relatively small number focused on the primary constituent of the IVD, i.e., the extracellular matrix and its collagen content. Several concerns exist over the direct application of these results to cervical discs—extrapolation of lumbar results, utilization of surrogates for normal human discs (e.g., adjacent discs obtained during surgery in symptomatic individuals or for deformity indications), age heterogeneity, undisclosed disc region (e.g., anterior or posterior AF) and analytical problems resulting from the use of “semi-quantitative” methods are just some of them [3–6]. Therefore, in this study we describe and compare the morphology, ultrastructure and collagen content of cervical discs from presumably asymptomatic young (under 35 years) and elderly (over 65 years) individuals. Our hypotheses are: 1) disc ultrastructure and collagen content are significantly modified during normal aging and 2) these modifications impact anterior and posterior disc regions differently.

## Material and Methods

Thirty C4-6 vertebral blocks were collected from unselected autopsies of recently-deceased (<6 hours) cadavers at the SVOC-USP. This study was reviewed and approved by the ICB-USP IRB (811/2007). Next of kin provided consent and were interviewed to exclude cadavers with known history of neck or back pain, neoplasms or rheumatological conditions as previously described [7]. In order to allow for degenerative changes to accumulate in the elderly group, a relevant time interval should separate both groups—ten years is the minimal amount demonstrated to cause a significant accumulation of these changes [8]. Here we arbitrarily defined 30 years as the interval: therefore, Group 1 (**G1**) included 15 cadavers younger than 35 years old and Group 2 **G2**), 15 cadavers aged 65 or older (Table 1). Throughout the study, C4-5 and C5-6 discs were analyzed jointly, thus resulting in 30 discs/age group. Specimens were assigned random identifiers and masked to researchers.

**Table 1:** Cadaver data: average +/- standard deviation.

	G1	G2	*p*
Age (yrs)	31.8 +/- 2.6	78.1 +/- 7.8	<0.001
Height (cm)	172.6 +/- 8.0	166.0 +/- 9.4	0.07
Weight (kg)	72.5 +/- 14.7	68.4 +/- 22.0	0.06
Male:Female	16:4	13:7	---

*p*, Student’s T analysis of **G1** versus **G2**.

### MR imaging

The IFUSP 1.5T MR scanner (Philips S15/ACSII, Netherlands) was employed to obtain T2 mid-sagittal and 2-mm axial images through the level of the C4-5 and C5-6 discs of five cadavers (ten discs) from **G1** and **G2** each. MR parameters were adapted to our specimens to replicate a T2 sequence (matrix = 512x225, TR/TE = 5000/130ms and FOV = 140x140mm). Specimens were placed in a tray, surrounded by air and scanned at room temperature (20–23 degrees Celsius). Discs were analyzed semi-quantitatively with a modified Okada grading system: individual scores (0, 1 or 2) were added and resulted in a final grade 0 (least) to 6 (most degenerated)[9]. **G1** and **G2** results were compared with the Mann-Whitney test (GraphPad Prism 6, San Diego, CA). A significance level of .05 was utilized throughout the study.

### Morphological grading

Following fixation in 4% formaldehyde for six months, all specimens were sectioned in the mid-sagittal plane and graded semi-qualitatively with the Thompson scale [10]. Degeneration was graded 1 to 5 and a **G1** *versus* **G2** comparison made with the Mann-Whitney rank-order test.

### Light microscopy

Discs and their intact endplates were decalcified in 0.25M EDTA for 30 days, followed by 1M EDTA for 5 days immediately before processing. Cervical discs were divided in the axial plane at their mid-point in two sectors, *anterior* and *posterior*. The NP was not visible to the naked eye in most cases. Fragments were frozen-sectioned on a sagittal (20 discs/group) or coronal (10 discs/group) orientation. Semi-serial, 8 μm sections stained according to hematoxylin-eosin (HE), Sirius Red (SR), Verhoeff’s iron-hematoxylin (mature elastic fibers) and Weigert’s resorcin-fuchsin (elastic and elaunin fibers) techniques [11]. Photomicrographs were acquired under normal and polarized (Sirius Red) light.

### Scanning electron microscopy (SEM)

Six discs from **G1** and **G2** each were randomly selected for SEM. Following decalcification and sectioning as described, the clean-cut mid-point surface of each specimen was attached, face-up, to an SEM stub [11]. Anterior and posterior cervical blocks from each disc were dehydrated (45°C for 12 hours), gold-coated and analyzed in a scanning electron microscope (Leo 435 VP, Cambridge, England).

### Collagen immunohistochemistry (IHC)

Six discs from **G1** and **G2** each were randomly selected for this experiment. A commercially-available ABC kit was utilized (ImmunoCruz ABC, SantaCruz Biotechnology, California). Eight μm-thick sections of each disc sector (*anterior* and *posterior*) were sequentially prepared according to manufacturer’s instructions—protease-based antigen unmasking (Table 2 - 30 minutes, 37°C), neutralization of endogenous peroxidase (1% H_2_O_2_ in PBS, 5 minutes), blockage of non-specific sites (1.5% blocking serum, 30 minutes), incubation with primary (12 hours, 4°C) and secondary antibodies (30 minutes, 37°C) and avidin-peroxidase conjugate (30 minutes, 25°C). DAB chromogen was allowed to react for 3 minutes and slides assembled in usual manner [6]. Each step was preceded by two PBS washes.

**Table 2:** Antibodies and proteases utilized in the study.

Antigen	Protease	Antibody data
Collagen I	0.2% trypsin	Abcam, ab90395
Collagen II	0.4% pepsin in 0.01N HCl	Santa Cruz, sc-59958
Collagen III	0.4% pepsin in 0.01N HCl	Sigma-Aldrich, C7805
Collagen IV	0.4% pepsin in 0.01N HCl	Sigma-Aldrich, C1926
Collagen V	0.4% pepsin in 0.01N HCl	Millipore, MAB3393
Collagen VI	0.4% pepsin in 0.01N HCl	Santa Cruz, sc-47712
Collagen IX	0.4% pepsin in 0.01N HCl	Millipore, MAB3304
Collagen X	0.4% pepsin in 0.01N HCl	Sigma-Aldrich, C7574

Primary antibodies against human collagen types I, II, III, IV, V, VI, IX and X were utilized. Single- (no primary antibody) and double-negative (no antibody) controls were included. Quantification was performed with an area-based method as previously described and expressed as a percentage of total area [12]. Ten random microscopy fields at 1000x magnification from each disc sector were quantified, resulting in a total of 60 data entries per disc sector, per group. Expression of each collagen type was analyzed against the negative controls with one-way ANOVA and *post-hoc* Tukey tests to determine if it was significantly different than background or non-specific binding. Comparisons between disc regions (*anterior* versus *posterior*) and groups (**G1** *versus* **G2**) were performed with T test. Staining unable to be differentiated from artifact was only included in the **G1** versus **G2** comparison and excluded from sector and segment analyses. No adjustments were made for multiple comparisons as all modifications in collagen staining were considered part of the same pathophysiological process and analytical priority was given to the general picture over any individual comparison [13].

## Results

### Magnetic resonance imaging

**Fig 1:**
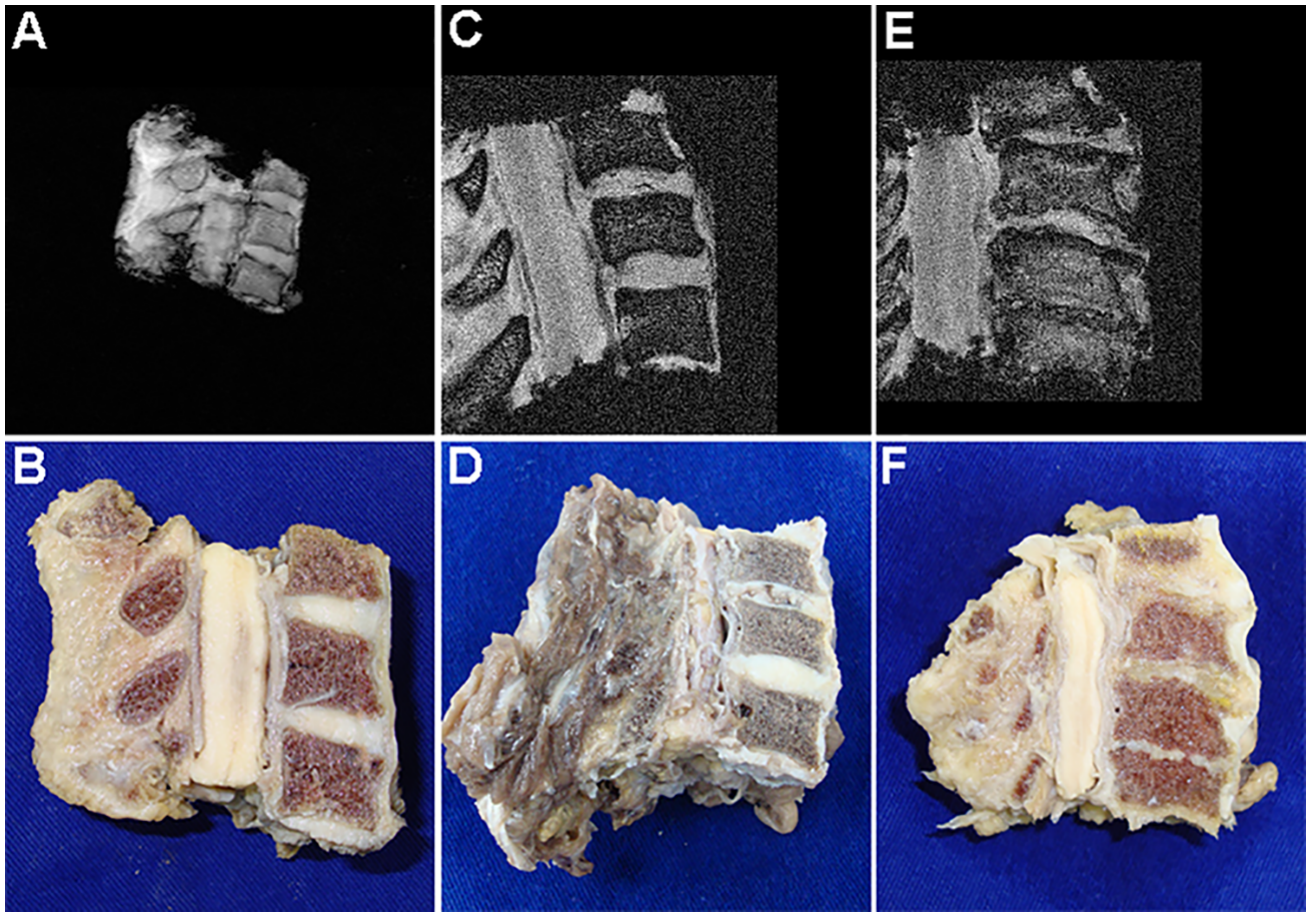
MR and corresponding mid-sagittal views of cervical vertebral blocks. **G1** specimen with only incipient degenerative findings is demonstrated in **A** and **B**. Advanced degeneration was manifested in **G1** primarily through horizontal tears such as seen in the C4-5 disc of this another **G1** specimen (**C** and **D**). Complete disc collapse was present in over 50% of **G2** specimens as seen here (**E** and **F**). The C3-4 disc was included in this last specimen but not graded.

**Fig 2:**
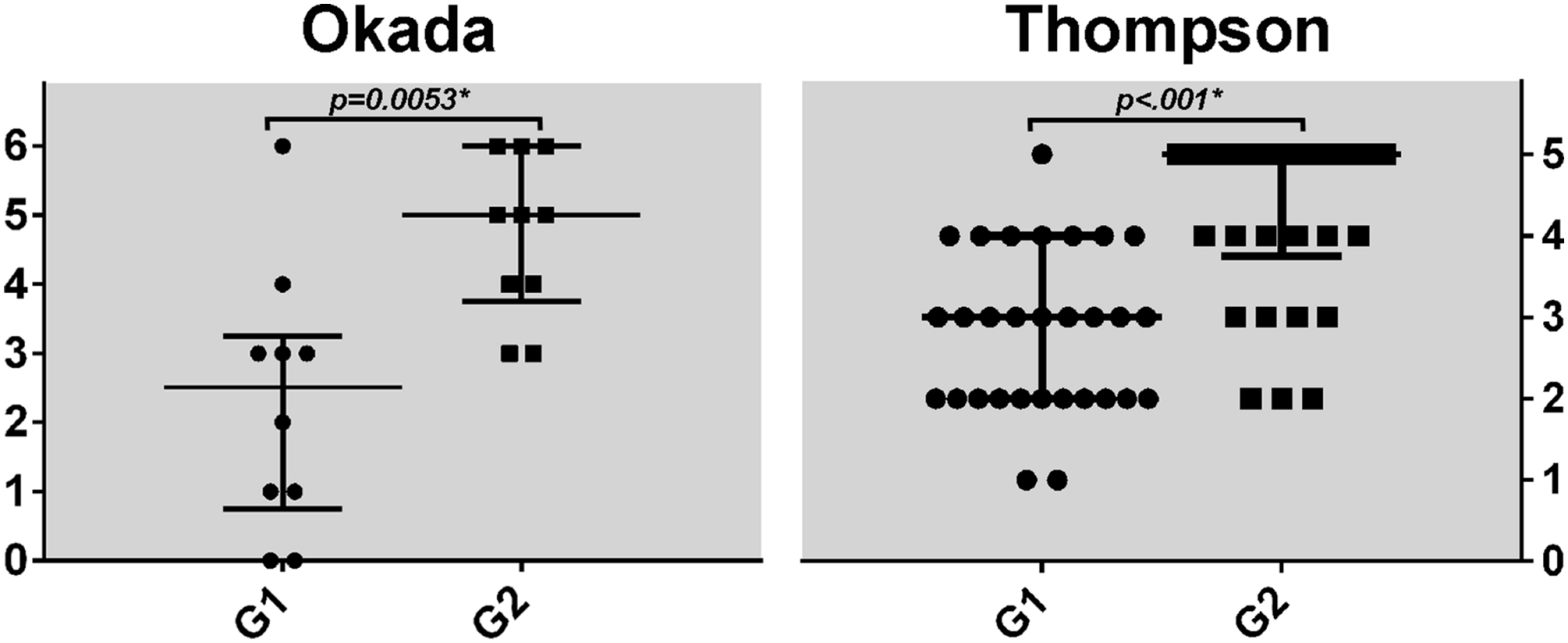
Distribution of Okada (n = 10) and Thompson (n = 30) scores per group. Bars represent median and interquartile range. Comparison performed with Mann-Whitney test.

Cervical discs from **G1** were not devoid of degenerative findings, though these were generally incipient (Figs 1 and 2). The most common finding in **G1** was simply the loss of AF-NP distinction; when more advanced findings were seen, these were mostly horizontal tears. These tears were usually confined to only one of the two discs in each segment, occasionally leading to widely different Okada scores within the same cadaver. In **G2**, advanced degeneration was usually manifested as complete disc collapse (Fig 1), which typically impacted both graded discs in a similar manner. Semi-qualitative grading confirmed progression of degenerative features from **G1** to **G2** as expected (*p* = .005 - Fig 2).

### Morphological grading

Although Thompson grades 1 through 5 (Th1 –Th5) were represented in our samples, Th1 discs were rare in **G1** (2/30) and non-existent in **G2** (Fig 2). Since identification of a distinct nucleus is necessary for grades Th1 and Th2, even Th2 discs were rare in **G2** (3/30). Advanced degeneration in **G1** was usually due to small osteophytes and fissures but not complete ankylosis—accordingly, 7 of 30 discs were graded Th4 but only 1 received a Th5 grade. Tears could also affect the two graded discs of the same cadaver differently as seen in Fig 1C and 1D but the difference in degeneration was always restricted to one Thompson grade. Accumulation of degenerative features from **G1** to **G2** was also confirmed through semi-quantitative grading (*p* < .001 - Fig 2).

### Light microscopy

**Fig 3:**
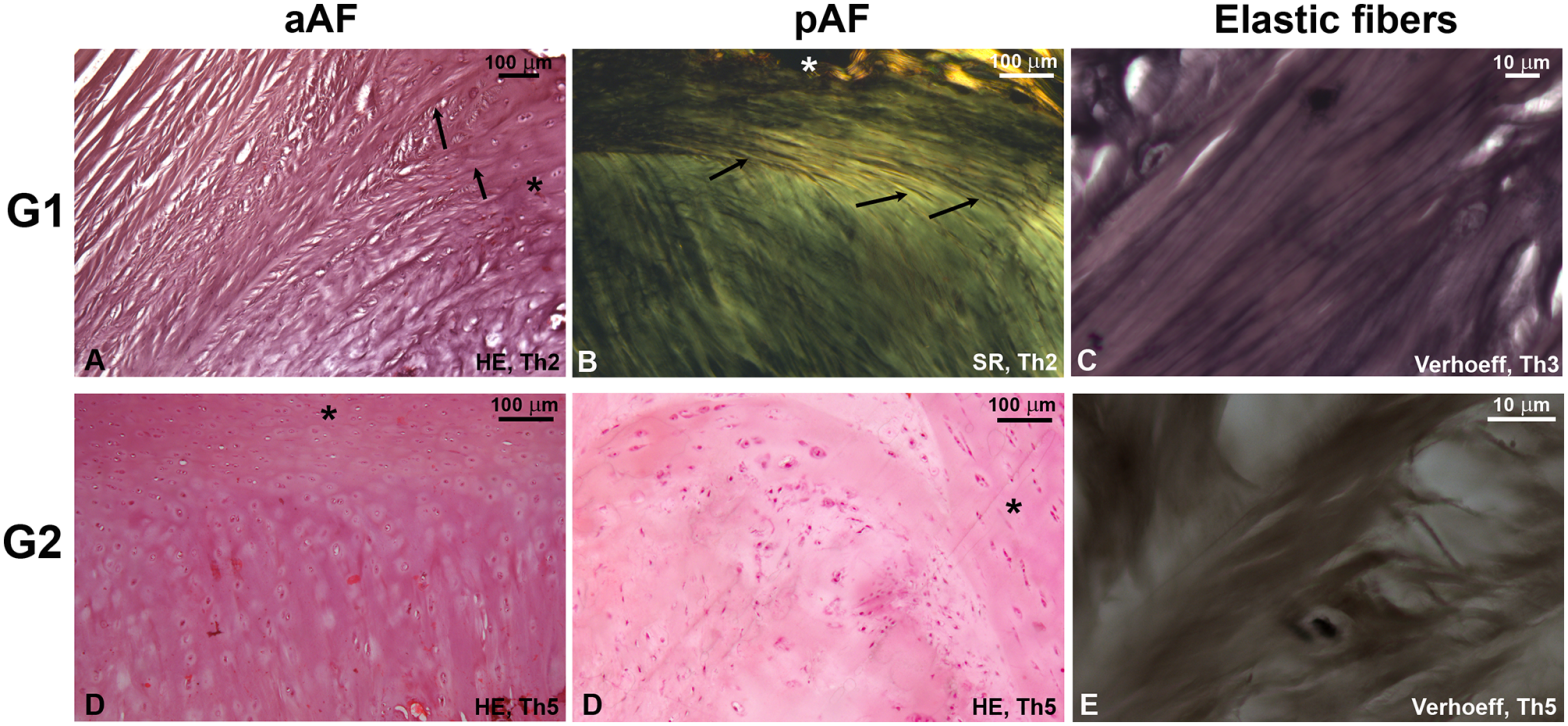
Light microscopy slides. A well-delimited structure of fibrous lamellae in both anterior and posterior AF of **G1** discs is substituted for a degenerated phenotype in **G2** with compact, fibrocartilaginous matrix populated with numerous chondrocyte clusters. Sharpey-type insertion of AF fibers (arrows) into the endplate (asterisks) is well visualized in **G1** discs and maintained in **G2**. In the anterior AF, fibers insert directly into the endplate while in the posterior sector this insertion is at an obtuse angle (arrows). Elastic fibers were rarely found in **G2** discs, regardless of Thompson (Th) score. SR, Sirius Red. HE, hematoxylin and eosin.

A morphologically “young” fibrocartilaginous phenotype consisted of an anterior AF (aAF) with dense alternating lamellae, predominantly longitudinal in more superficial areas and oblique in deeper sectors, and a thinner posterior AF (pAF) with predominantly longitudinal fibers (Fig 3). Even in **G1**, a central area of discernible loose connective tissue was seen in only 2/30 cervical discs; the usual finding was a central area of denser fibrous tissue with chondrocyte clusters in its interior. Under polarized light, the AF lamellae can be seen to firmly insert themselves into the adjacent endplates in the manner of Sharpey’s fibers—regardless of how degenerated the disc was, the central area never exhibited these insertions. Elastic fibers were found aligned with the connective tissue lamellae in the AF and staining was concordant between the Verhoeff and Weigert techniques, suggesting these fibers are of the mature elastic type. Elastic fibers were found in smaller quantities in more degenerated **G1** specimens but were still present.

A morphologically “elderly” phenotype seen in most **G2** discs included marked disruption of the lamellar structure, endplate hypertrophy and more numerous and larger chondrocyte clusters within the disc, while SR staining revealed a substitution of green-refringent fibers for opaque material. With the exception of a single cervical disc, **G2** specimens uniformly lacked elastic fibers regardless of Thompson score.

### SEM

**Fig 4:**
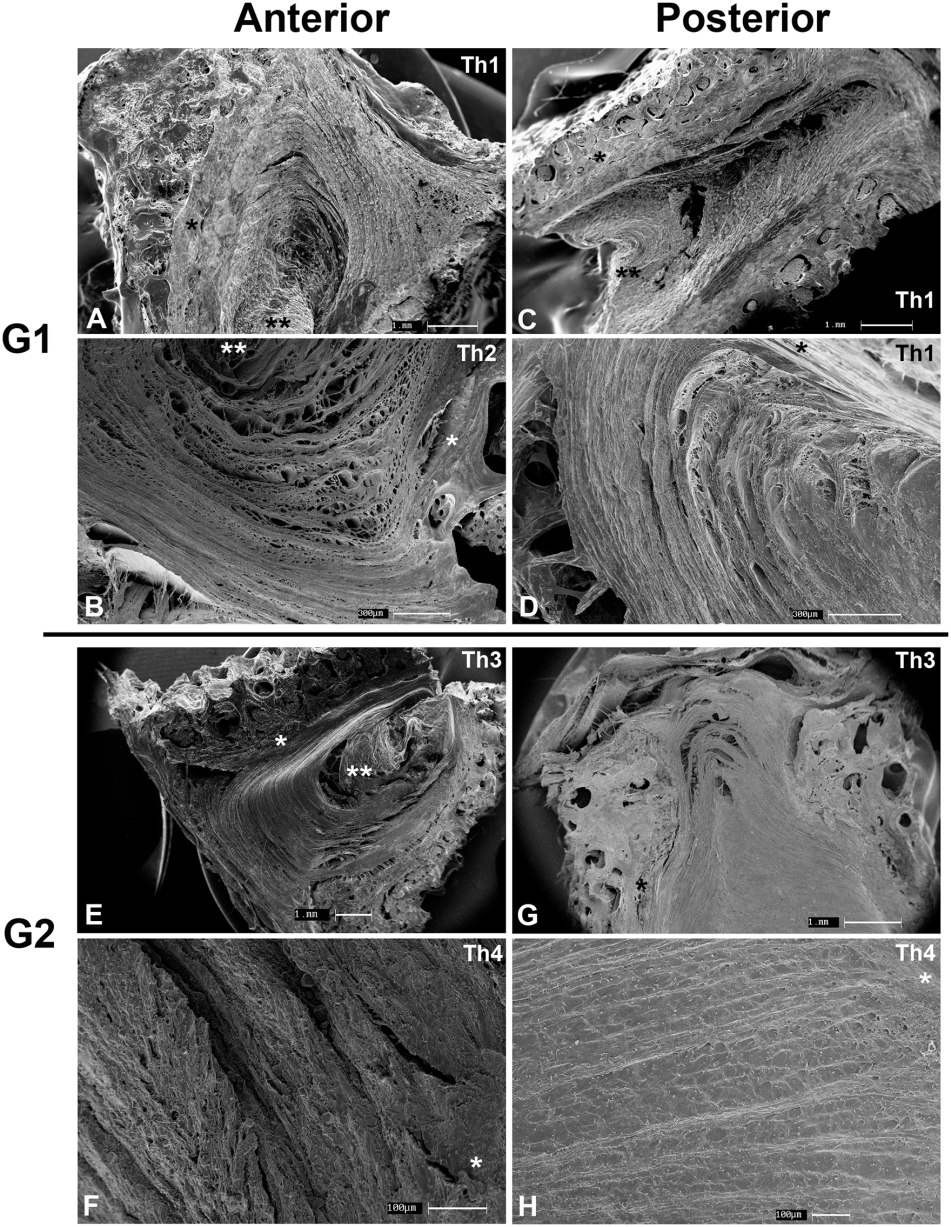
Scanning electron microscopy. **G1** (**A-D**) and **G2** (**E-H**) cervical specimens. AF of **G1** composed of alternating lamellae while longitudinal bundles predominate in the posterior AF. Insertion of the AF into the endplate (*) is perpendicular in the anterior AF and obtuse in the posterior AF. A central area of loose connective tissue without endplate insertion is present in both **G1** and **G2** specimens (**). Th, Thompson grade.

The superficial layers of the anterior and posterior AF were indistinct from the anterior (ALL) or posterior (PLL) longitudinal ligaments, while the pattern of intertwined lamellae predominates in deeper areas (Fig 4). A fine diagonal mesh can be seen between **G1** lamellae. Endplate insertion was perpendicular in the anterior AF and obtuse in the posterior AF. SEM was the only method that could visualize the NP as a separate structure in all specimens due to its lack of lamellar organization and endplate insertion and greater retraction due to higher water content than the AF, regardless of the extent of degeneration. In **G2** specimens, the AF matrix was far denser resulting in little space between lamellae, with preserved endplate insertion.

### Collagen immunohistochemistry

**Fig 5:**
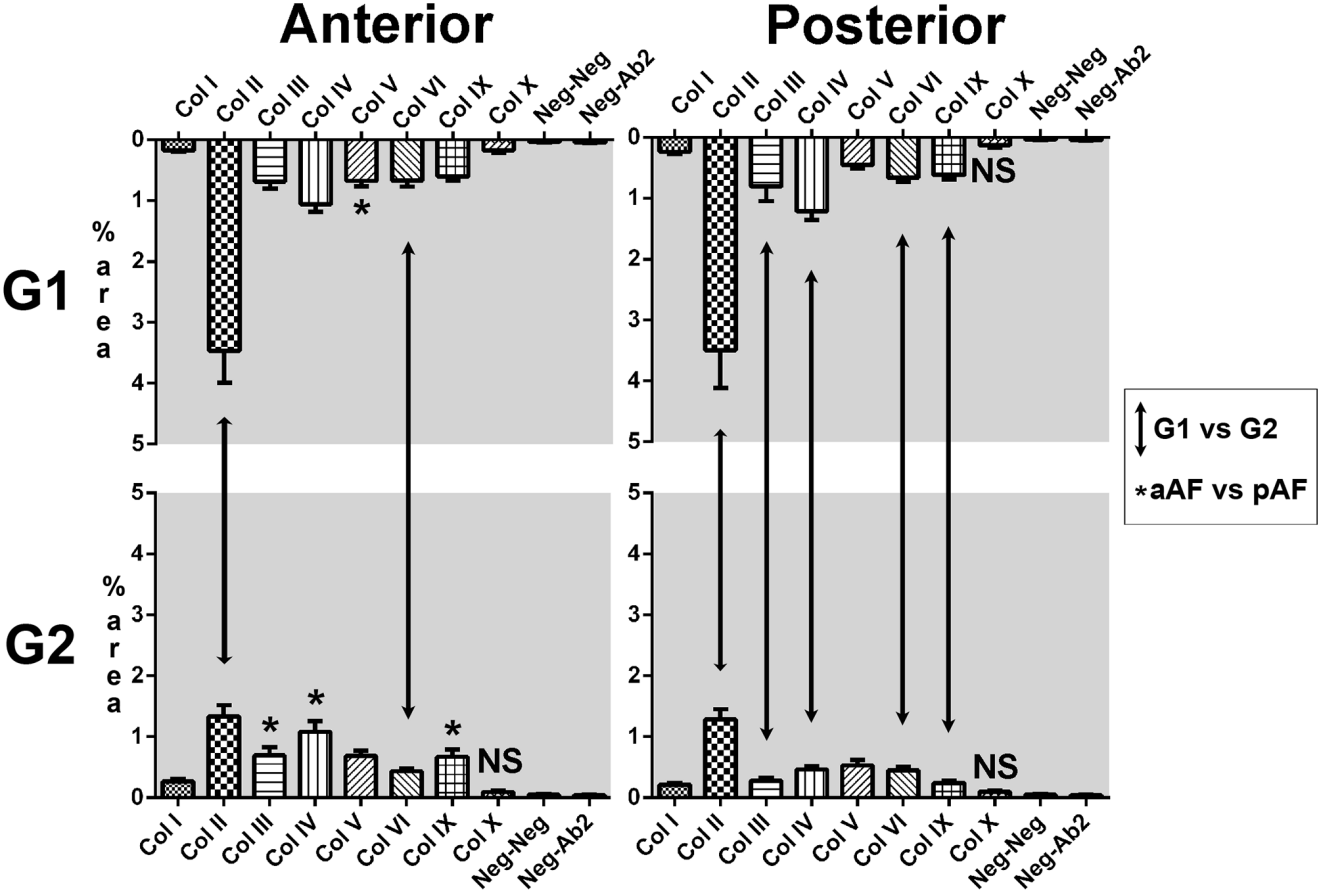
Stained area per collagen type (%). Predominance of collagen type II in **G1** and an approximate 60% reduction in **G2**. Vertical arrows indicate *p* < .05 in **G1** vs. **G2** comparison; black asterisks mark *p* < .05 in *anterior* vs. *posterior* comparison and are located in the frame with the higher expression. NS, staining not significant. Error bars represent standard error of the mean.

**Fig 6:**
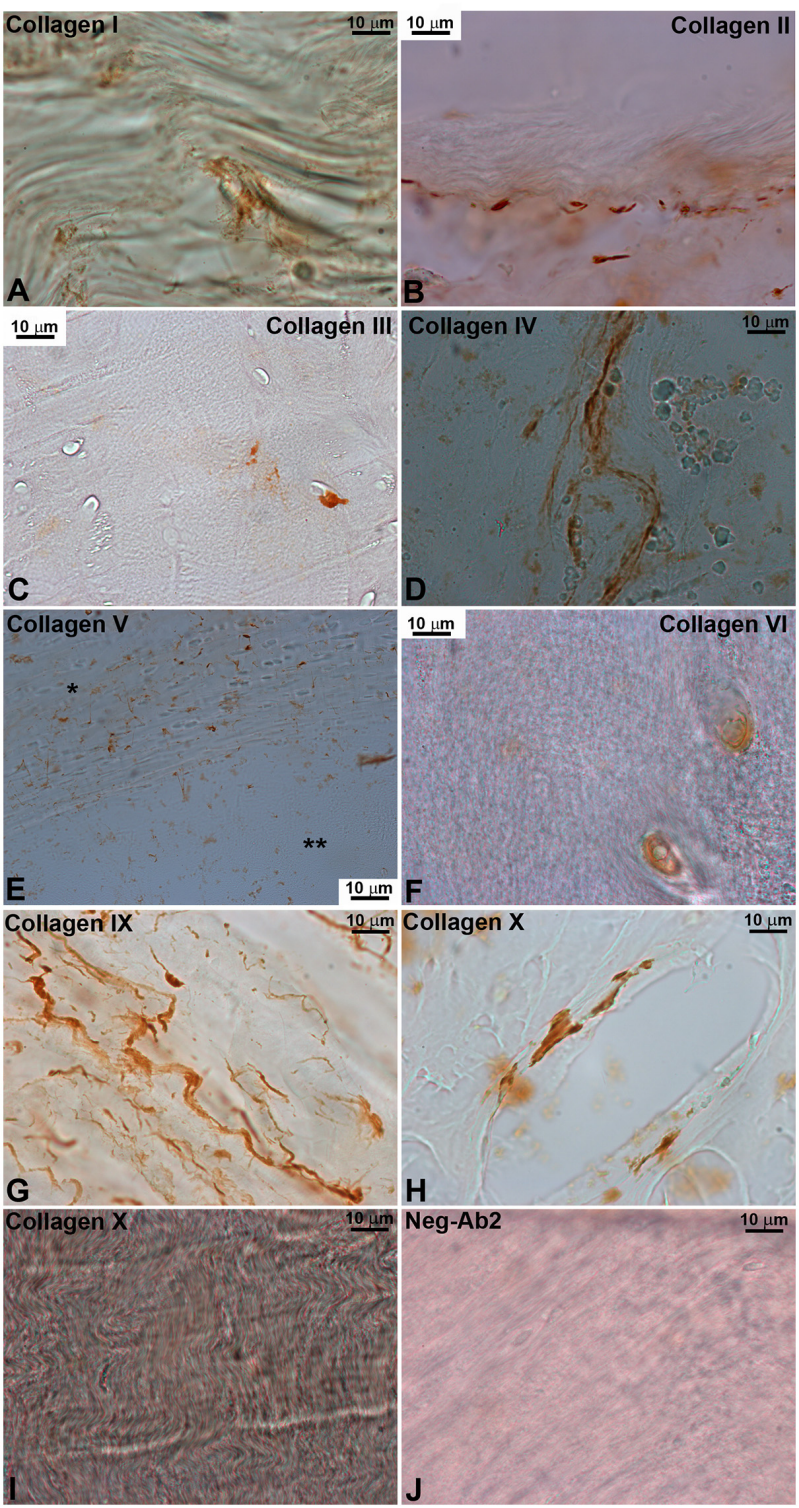
Immunohistochemistry of the different collagen subtypes. Two main patterns were seen: extracellular, filiform reactivity with collagens I, II, IV, VI and IX, and intra-/peri-cellular reactivity with collagens III, V and VI. Staining of collagen X was not significant in most areas but a rare positive example is included with filiform aspect (**H**). Endplate (*) and disc (**) are shown in **E**; detection of collagen V was higher in the endplate. Neg-Ab2, negative control with secondary antibody only.

All tested collagen types stained an area larger than that stained in either negative controls in every disc sector in both **G1** and **G2** with the exception of collagen X (Fig 5). Qualitative analysis demonstrated two main staining patterns. Collagens I, II, IV and IX had predominantly extracellular, filiform reactivity within the structural bundles composing the lamellae of the AF (Fig 6). Intra- and peri-cellular reactivity was seen with collagens III, V and VI, although collagen V was mainly present next to the vertebral endplates. The area stained by collagen X antibodies was not significantly different than the negative controls except in the anterior AF of **G1** discs; when present, its expression was similar to the filiform pattern.

Overall, collagen reactivity in **G2** was greatly decreased when compared to **G1**. Collagen II was the most common collagen in both young and elderly discs but its expression was reduced by approximately 60% in **G2**. Collagen VI was also reduced in both anterior and posterior sectors from **G1** to **G2**, while types III, IV and IX were reduced in the posterior sector only. Regional differences (*anterior* vs. *posterior*) were rare and usually small; in **G1**, collagen V was seen in a larger area in the anterior disc while in **G2** the expression of collagens III, IV and IX was higher in the anterior disc as well.

## Discussion

The morphological and collagen profile modifications described above are the phenotypical and ultrastructural manifestations of a multifactorial process generically described as intervertebral disc degeneration. Based on the observation that endplate vascular channels close in the first two decades of life, a very early theory was conceived in that IVD degeneration was secondary to decreased availability of oxygen and nutrients to the cells of the adult disc, further substantiated by description of biochemical pathways that mimic changes in the proteoglycan structure of the disc [6,14–18]. How the collagen content is modified is less clear and a relatively more recent research interest. Axial and torsional stresses have been shown to play a role as well as the modulating actions of fibroblasts, chondrocytes and even structural molecules such as collagen IX. Also less clear is whether this process occurs in the same manner in cervical discs—since axial and torsional loads are different in relation to the lumbar spine, it would be natural to suppose that degeneration is different too [19,20]. Only a small fraction of all research generated on disc degeneration is based on cervical material, despite the clinical importance of neck pain [21]. A possible explanation for this fact is that lumbar discs naturally yield more material for analyses due to their larger size, which may be important when dealing with small animal models. In any case, there is no reason to assume disc degeneration occurs in the same manner in the cervical spine—despite a common embryological origin, anatomical and functional differences are well-known [5]. The same principles apply to the aAF and pAF in cervical discs—not only the anatomy is different, but the loads and intradiscal pressures borne by the aAF and pAF are markedly different [5,22].

Our morphological, MR and histology data largely confirms literature data that degenerative findings can be found in young individuals and are significantly increased in older, asymptomatic individuals. Boden *et al*. demonstrated in the early 1990s that disc degeneration may be seen in MRs of approximately 60% of asymptomatic subjects in their 60s; these numbers have increased into the 85–90% range as MR technology improved. Asymptomatic progression of these changes over a long period has also been demonstrated in upwards of 80% of individuals from a Japanese cohort [8,23]. These numbers probably reflect a limitation of the examining method rather than the existence of naïve discs in late adulthood. Christe *et al*. have already shown that MR vastly underestimates the extent of degenerative alterations in cervical discs. This is corroborated by our findings regarding the cervical NP, which through SEM was demonstrated to persist in older discs as a separate structure although a clear boundary was not seen through MR or light microscopy [24]. It is rare, however, that a disc would be considerably more degenerated than the rest of the segment in the same individual—Thompson scores never differed by more than one grade, which validates our decision to use C4-5 and C5-6 results jointly in statistical analyses and support diagnostic and treatment algorithms that consider discs with dissonant degeneration (when compared to other discs in the same individual) as targets for therapeutic intervention [25].

Light microscopy results demonstrate the loss of the loose lamellar arrangement in the AF and substitution by a more compact arrangement with numerous chondrocyte clusters. Although these are findings described a long time ago in the lumbar spine, only recently Sitte *et al*. have demonstrated it in the anterior part of cervical discs [26]. The Sharpey-type insertion of aAF fibers into the endplate as well as the obtuse manner in which it takes place in pAF had not been described before in the human cervical spine. These may represent a consequence of the lordotic configuration of the cervical spine—ideally this would be investigated utilizing discs of neonates, before a lordotic curvature is developed but this material is even more difficult to obtain. To the extent of our knowledge, it is also the first time that the fiber arrangement of cervical AF is studied with SEM, also demonstrating the Sharpey-type insertions and substitution of the loose matrix of the AF by a much more compact one with predominantly cartilaginous characteristics.

Collagen profiling revealed that type II collagen is the most common in the cervical disc, followed by collagen IV. Detection of all collagen types was decreased in **G2** but in the aAF only types II and VI reached statistical significance, while in the pAF types I, II, IV, VI and IX were significantly decreased. A single regional difference was seen in **G1** (collagen V) but in **G2** those types that did not yet reach a significant reduction in the aAF (types III, IV and IX) accounted for the significant regional differences between aAF and pAF. Rather than demonstrate a different degeneration pattern, these results probably reflect the same process occurring faster in the pAF than in the aAF. It further suggests that the load each disc sector is subjected to alters its collagen content, as suggested by Brickley-Parsons et al. in young scoliotic spines [19].

Regarding the contribution of each collagen type to disc structure, there is no comparable data for human cervical discs in the literature; lumbar data utilizing semi-quantitative or chromatography methods pointed towards a predominance of collagen II in the interior of the lumbar disc with less significant components of collagens III (in the AF), V, VI and IX (diffusely)[6,27,28]. Collagen II as the main type within the disc had been first suggested by Eyre and Muir in the lumbar spine of suids and further demonstrated in humans of different ages [29–31]. Eyre and Muir described collagen II as making up at least 85% of all collagen in the lumbar spine at different ages but only analyzed types I and II using CNBr digestion and electrophoresis [31]. Previous studies utilizing immunohistochemistry had significantly different results—for example, Nerlich et al. describe type III as more frequent in the young NP—and it is the first time Eyre and Muir’s results are replicated with an area-specific method. Collagen IV expression was thought previously to not occur in most age ranges—Nerlich *et al*. had suggested expression could happen in advanced degeneration [6]. Several methodological differences may account for these different results—different vertebral region, quantitative method, utilization of surrogates for normal discs (such as samples from scoliotic patients in Beard *et al*.[27]–so our results are actually in agreement with the vast majority of published collagen literature in the human IVD, although widely different than the biggest study utilizing the same method [6,31,19].

There are a number of limitations to our study. Firstly, despite the screening interview, there is always a possibility the studied individuals were indeed symptomatic. The rarity of adequate human material is the biggest impediment to properly study morphological alterations in asymptomatic individuals and there is no truly equivalent bipedal animal model [32,33]. This single reason may be responsible for several of the deficiencies of prior studies. On the other hand, short of prospectively enrolling a massive number of individuals and waiting decades for their deaths, we see no better alternative to this model and it has been employed in numerous studies [7]. Secondly, when this study was designed we imagined that degeneration would affect not only disc regions but also vertebral segments differently (hypothesis #2). Therefore, we tried to analyze as many disc regions as possible and that may have resulted in a seemingly small number of studied samples but in fact this corresponds to the largest study of IVD material with SEM and IHC in human specimens [34–36]. Once results were analyzed, it was satisfactorily demonstrated that the extracellular matrix of cervical discs is significantly modified during normal aging (proving hypothesis #1) but we realized the degenerative process is very similar in both the aAF and pAF and thus we are unable to prove hypothesis #2. We would thus favor analyzing fewer disc regions in future studies while increasing the number of repetitions per experiment.

## Conclusions

The results presented here are relevant to dispel the notion of a “pathologic” collagen marker within cervical discs and to delineate a structure which is not only at the center of a common health problem but is also the object of number of restorative strategies [6,36,37,38]. Disc ultrastructure is similar regardless of area (anterior or posterior) and collagen profile is significantly modified during aging, mainly by a significant reduction in collagen content and a reduction in the relative content of collagen II. Therapeutic modification of disc degeneration, whether drug-, cell- or tissue engineering-based, will ultimately either arrest the process leading to the modifications here described or emulate its mechanical properties.
